# Monitoring the Path to the Elimination of Infectious Diseases

**DOI:** 10.3390/tropicalmed2030020

**Published:** 2017-06-26

**Authors:** John M. Drake, Simon I. Hay

**Affiliations:** 1 Odum School of Ecology, University of Georgia, Athens, GA 30602-2202, USA; 2 Center for the Ecology of Infectious Diseases, University of Georgia, Athens, GA 30602-2202, USA; 3 Department of Zoology, University of Oxford, Oxford OX2, UK; 4 Institute for Health Metrics and Evaluation, University of Washington, Seattle, WA 98121, USA; sihay@uw.edu; 5 Big Data Institute, Li Ka Shing Centre for Health Information and Discovery, University of Oxford, Oxford OX3 7LF, UK

**Keywords:** bifurcation delay, critical slowing down, elimination, endgame, smallpox

## Abstract

During the endgame of elimination programs, parasite populations may exhibit dynamical phenomena not typical of endemic disease. Particularly, monitoring programs for tracking infection prevalence may be hampered by overall rarity, the sporadic and unpredictable timing and location of outbreaks, and under-reporting. A particularly important problem for monitoring is determining the distance that must be covered to achieve the elimination threshold at an effective reproduction number less than one. In this perspective, we suggest that this problem may be overcome by measuring critical slowing down. Critical slowing down is a phenomenon exhibited by nonlinear dynamical systems in the vicinity of a critical threshold. In infectious disease dynamics, critical slowing down is expressed as an increase in the coefficient of variation and other properties of the fluctuations in the number of cases. In simulations, we show the coefficient of variation to be insensitive to under-reporting error and therefore a robust measurement of the approach to elimination. Additionally, we show that there is an inevitable delay between the time at which the effective reproduction number is reduced to below one and complete elimination is achieved. We urge that monitoring programs include dynamical properties such as critical slowing down in their metrics for measuring achievement and avoid withdrawing control activities prematurely.

## 1. Introduction

Eradication of an infectious disease is the ultimate success in public health [1]. Smallpox is the only infectious disease of humans to have been eradicated to date [2]. Significant, albeit fragile, progress has been made toward the eradication of polio [3], dracunculiasis [4], and yaws [5]. Moreover, local elimination plans are ongoing for malaria [6], lymphatic filariasis [7,8], measles [9], rubella [10], onchocerciasis [11,12], schistosomiasis [13], and trypanosomiasis [14].

Epidemiology, surveillance, and the changing effectiveness of control actions all present unique “end game” challenges in eradication and elimination efforts [1,15]. A particularly intractable problem has been maintaining the political will to continue interventions as the number of new cases declines, partly because of the declining marginal returns on investment when measured in terms of the reduction in cases and partly because of the difficulty of monitoring sparsely occurring disease episodes among largely inaccessible populations [14,16,17]. If surveillance systems for these diseases could identify the approach to ultimate elimination, in contrast to merely marginal declines in the number of new cases, philanthropic funders, public health officials, and others might be better armed to both justify and target efforts during the “longest last mile,” especially if it can be shown that the tipping point has been crossed and all that remains is to sustain the gains already achieved and allow transmission to run its course [18,19,20].

Here we propose a new approach to monitoring the path to elimination, based on recent developments in the theory of leading indicators for dynamical transitions [21]. Leading indicators are statistical patterns exhibited by certain complex systems as they approach a tipping point between alternative states, primarily due to *critical slowing down*, in which the tendency for a dynamical system to return to its steady state is increasingly weakened [21]. In epidemiology, endemism and disease-free status are alternative states, with the vaccination threshold acting as the tipping point between [22]. Although not all switching among alternative states is preceded by leading indicators [23], the transcritical bifurcation that occurs in disease elimination does, implying that the sequence of case reports will contain statistical signatures of the approach to elimination [22,24]. Thus, leading indicators based on critical slowing down may provide a means to measure how successful elimination efforts have been, particularly by indicating if the tipping point has already been crossed or is close at hand [24].

Distinguishing these conditions is clearly important for policy. If the threshold has already been crossed, elimination only requires that the current policy be maintained while transient chains of transmission are allowed to peter out; alternatively, if the threshold is near but not yet crossed, then increases in effort or pulse interventions such as supplementary vaccination campaigns may be required to push the contagious process over the tipping point. Our previous studies developed the theory of critical slowing down in disease transmission systems [22,24], but did not address practical issues such as under-reporting or extrapolating from measured signals to predict the time of threshold crossing.

Smallpox illustrates the potential of this theory for monitoring progress toward disease elimination. Smallpox is an acute and often lethal infection, and was globally eradicated in the wild in 1977 [2]. Nation states differed in both the delivery of vaccination and the speed of elimination, but it was frequently on the time scale of decades, with a rapid decline followed by a long tail. Presumably, the vaccination threshold was crossed sometime before or during the decline phase. The simple question is, when?

## 2. Model

**Figure 1:**
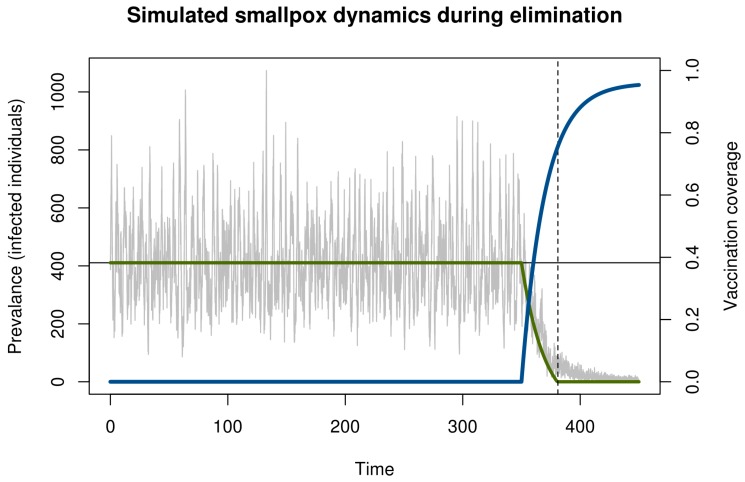
Simulation of smallpox elimination through vaccination. The gray line shows the number of infected individuals over time. The blue line shows vaccination coverage, which approaches a maximum at 0.96. The green line shows the deterministic equilibrium that would be achieved if the vaccination rate was held constant and the time-dependent value. Disease dynamics are given by a stochastic SIR model with states *S*, *I*, and *R* for the number of susceptible, infected, and immune persons, respectively; mean field equations d*S*/d*t = μ*(*S + I + R*)(1−*ρ*) − β*SI*/(*S* + *I* + *R*) – ξ*S* − *μS,* d*I/*d*t =* β*SI*/(*S* + *I* + *R*) + ξ*S* − *μI* − γ*I,* and *dR/dt =* γ*I* + *μ*(*S + I + R*)(*ρ*) − *μR*; and parameters for transmission *β* = *R*_0_(*γ* + *μ*) ≈ 121.7, recovery *γ* = 365/12 ≈ 30.4, demography *μ* = 1/60, externally acquired infection ξ = 0.001, speed of vaccination roll out *σ* = −0.05, maximum possible vaccination coverage *a* = 0.96, and time that vaccination begins *s* = 100. All rates are in units of years. The function *ρ*(*t*) = *a*(1 − exp[*σ*(*t* − *s*)]) is the time-dependent vaccination rate. Total population size in this simulation was 100,000 individuals. Solutions were obtained using the adaptive tau-leaping algorithm [25]. Parameters follow Ferguson et al. [26].

We illustrate critical slowing down in smallpox elimination with a simple model of transmission and vaccination (Figure 1). This model begins in the endemic state and evolves stochastically assuming a policy of newborn vaccination beginning in year 350. Vaccination programs are imperfect and require time to ramp up. To realistically represent the roll-out of a vaccination campaign, we assumed that disease prevalence and immunity at the start of the simulation was at the natural endemic equilibrium. Vaccine coverage started at zero and increased asymptotically toward 96% (Figure 1). The time the vaccination threshold was crossed is plotted as a vertical dashed line. As expected, before vaccination begins, the dynamics fluctuate around an endemic equilibrium. During the campaign, the number of cases declined, crossing the deterministic vaccination threshold in year 381. The pathogen was not eliminated until much later. This long tail is an example of *bifurcation delay*.

Both the approach to the tipping point (dashed line in Figure 1) and the transmission tail are of interest. From a health policy standpoint, the distinction between these two—and the ability to recognize which phase one is in—is key. If vaccination coverage can be maintained long enough once the threshold has been reached, then elimination is sure to occur eventually. By contrast, if the threshold has not been reached, then elimination is very unlikely and any stochastic extinction that may occur is in a fragile state that can be reversed at any time. During the time between crossing the threshold and elimination, it is important not to reduce vaccination pressure, since ceasing vaccination during this period would allow the disease to return to pre-threshold levels and the gains of the campaign will have been wasted despite having achieved the necessary coverage to ensure elimination.

**Figure 2:**
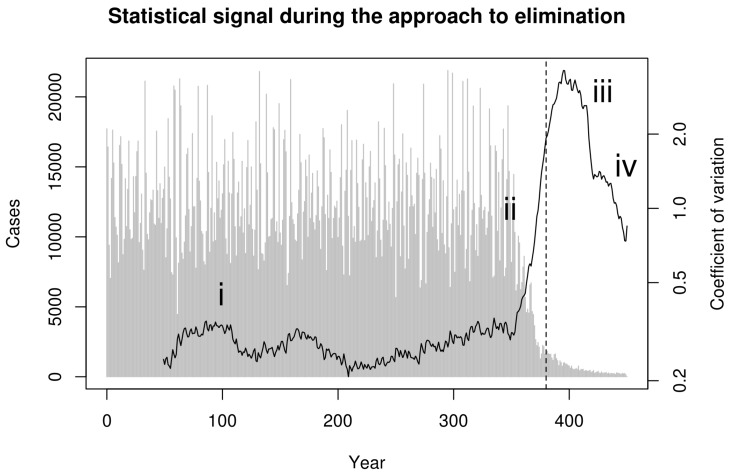
Statistical signal of the approach to the vaccination thresholds in simulated smallpox elimination. Contagion systems exhibit critical slowing down in the approach to a tipping point such as the vaccination threshold. The coefficient of variation in a moving window provides a measurement of the magnitude of this slowing down. We calculated this statistical signature by first detrending with a one-sided filter and then computing the coefficient of variation in a moving window of 30 observations. This figure shows this statistical signal to begin increasing with the onset of vaccination and to rise dramatically as the approach to the vaccination threshold (vertical dashed line) is approached.

Next, we consider whether these phases can be distinguished in data that can be derived from surveillance. To reflect real-world data collection, we first aggregated simulated cases to annual reporting intervals. Recent theoretical developments predict that the coefficient of variation in the number of cases in a moving window should be a sensitive and specific indicator of the approach to the vaccination threshold [22]. Indeed, calculating the coefficient of variation (CV; the ratio of the standard deviation to the mean) in a moving window over the annualized simulated cases in Figure 1 shows a characteristic dynamical pattern: (i) stationary fluctuations in *the pre-campaign era*, (ii) substantial and sustained rise in CV beginning with the start of the elimination campaign during the *approach to the vaccination threshold*, (iii) erratic fluctuations following the achievement of the vaccination threshold but *preceding elimination*, and (iv) a final decline as the pathogen approaches *complete elimination* (Figure 2).

**Figure 3:**
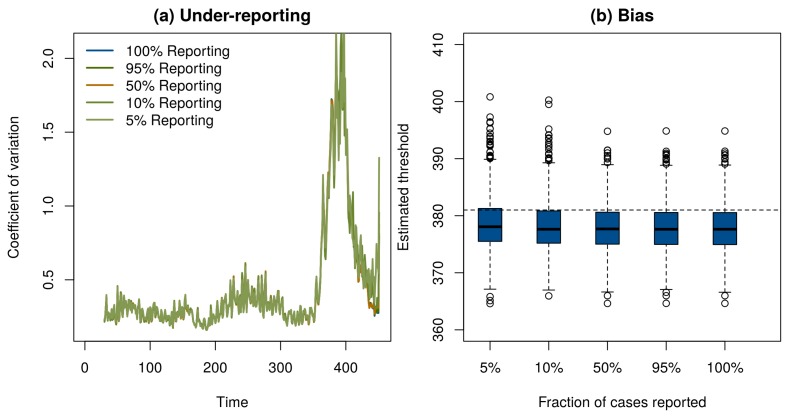
(**a**) Under-reporting had negligible effect on the ability of the moving window coefficient of variation to respond to the approach to the vaccination threshold. (**b**) A hyperbolic approximation was used to forecast the time the vaccination threshold would be reached. The estimated crossing time in 1000 simulations (boxplots) was on average slightly earlier than the true time of year 381 (horizontal dashed line). Under-reporting had little effect on the precision or bias of predictions.

Of course, if one has perfect case reporting, then it is simple enough to inspect the number of case reports over time to determine that the elimination campaign is continuing to realize results—although it is important to note that this will not tell us if the vaccination threshold has been reached. In order to determine if our method works with incomplete case reporting, we looked at case reports that were binomial samples of 95%, 50%, 10%, and 5% of the true number of cases in each year. Surprisingly, despite the fact that case reports differ by three orders of magnitude and themselves contain no information about the true prevalence in the population, the statistical signals indicating the approach to the vaccination threshold were practically indistinguishable (Figure 3a). This result shows that it may be possible to monitor the path to elimination by taking new approaches to processing surveillance data, even if they are subject to a significant amount of under-reporting. Details of these simulations are provided in online supplementary material.

These results show that critical slowing down accompanies the approach to the vaccination threshold and is detectable using very simple statistical tests. We also tested if the timing of the vaccination threshold itself is identifiable using these methods. Motivated by the theoretical prediction that the coefficient of variation would diverge at the vaccination threshold [22], we approximated the moving window signal with the hyperbolic equation *w_t_*= *a*/(*t* − *c*) + *b*, where *w_t_* is the calculated value of the signal at time *t*, *a* is a fit coefficient that governs the speed of divergence, *b* is a fit coefficient corresponding to the baseline value of *w*, and *c* is the location of the threshold. To test this model, we simulated 1000 realizations of the epidemic model in Figure 1. We then automated the estimation of the threshold by fitting *a*, *b*, and *c* by nonlinear least squares to data for each simulation from its start until the point it reached 75% of its maximum value. The estimated time that the threshold would be crossed was slightly biased and independent of the level of under-reporting (Figure 3b). We suspect the bias evident in Figure 3b is due to the approximation *w_t_*= *a*/(*t* − *c*) + *b*. Accordingly, we consider the observed bias (<1%) to be remarkably small. We consider it a high priority for further work to develop a more complete understanding of the conditions under which the time of threshold crossing can be accurately predicted.

## 3. Conclusions

In conclusion, these examples demonstrate two key properties of the dynamics of an infectious disease on the road to elimination. First, there is an inevitable delay between the time that the elimination threshold is achieved and the time that the last infected individual acquires infection. This is related to the phenomenon of *bifurcation delay*, described by Dibble et al. [27] in the context of disease emergence. Second, the statistical properties of fluctuations in the number of cases can indicate where along the path to elimination a particular population lies. In the approach to the vaccination threshold studied here, the coefficient of variation in a moving window increased continuously with vaccination pressure, providing a possible way to document gains from increases in effort. Areas for further research include determining what statistics—among dozens now available [28]—give both sensitive and specific warning of epidemiological transitions [22,24], and whether or not spatially explicit analogs of the approach taken here may be more powerful [29]. Additionally, it will be important to establish how well these indicators perform under the more complicated conditions that affect real elimination campaigns, including spatial heterogeneity, control programs that may be intermittent or vary in intensity, and seasonal forcing. Moreover, further theoretical research is needed to establish that critical slowing down will be detectable in parasites with more complicated life cycles (i.e., schistosomes and Guinea worm). The evidence so far—primarily from analysis of the near-critical dynamics of the Ross–MacDonald model—suggests that vector-borne diseases like malaria are predicted to display critical slowing down [24].

Regardless of further developments, immediate and simple insights could be derived from simple simulation studies on the frequency and accuracy of reporting needed to monitor the ongoing near-critical contagion systems of polio in places where it remains endemic.

## Supplementary Materials

The supplementary materials are available online at http://www.mdpi.com/2414-6366/2/3/20/s1.
