# Supplementary material for: Monitoring the Path to the Elimination of Infectious Diseases
Source: Trop Med Infect Dis. 2017 Jun 26;2(3):20. doi: 10.3390/tropicalmed2030020 (PMC6082106; doi:10.3390/tropicalmed2030020)
Supplement: Supplementary file 1 [file tropicalmed-02-00020-s001.pdf]

Supplementary Materials: Monitoring the Path to the Elimination of Infectious Diseases

John M. Drake and Simon I. Hay

Introduction

This document is the supplementary material to the paper "Monitoring the path to the elimination of infectious diseases". This document provides R code to reproduce the simulated data and all figures contained in the main text as well as additional information. The thesis of that paper is that the phenomenon of *critical slowing down* may be used to document the elimination of endemic infectious disease through vaccination or other interventions. Here we study the dynamics of a smallpox-like pathogen during a program of vaccine rollout and elimination. We provide methods for estimating eraly warning signals such as the divergence of variance as the critical threshold is approached and demonstrate their robustness to under-reporting. We then develop a method for estimating the time that the vaccination campaign crosses the critical point and compare the performance of this method with a simple extrapolation strategy. It is shown that critical slowing down provides a robust and accurate way to monitor the elimination of an infectious disease.

Setup

The analysis will require some functionality from Carl Boettiger's earlywarning package, which is available on Github at https://github.com/cboettig/earlywarning.

```
> require(earlywarning)
```

To reproduce the published figures, we require the following scheme of named colors.

```
> ose1 <- rgb(85, 108, 17, m = 255)
> ose2 <- rgb(160, 108, 17, m = 255)
> ose3 <- rgb(114, 132, 56, m = 255)
> ose4 <- rgb(137, 152, 87, m = 255)
```

Simulations

We study simulations parameterized to represent smallpox. From Ferguson et al. (Ferguson et al. 2003. Nature 426, 681-685) we have that smallpox is SEIR-like with 30% mortality. R_0 is between

4 and 10. There is a 12 day incubation period (E-stage) and 12 day infectious period (I-stage; 3 day prodromal stage and 9 day symptomatic period). If we ignore the E-stage, then recovery rate is about 30.5 per year ($(12/360)^{-1} \approx 30.417$). This yields a vaccination threshold of 0.75 to 0.9, which is also about right.

For the simulated scenario, vaccination rate is defined to be a nonlinear function of time. The following R function returns the vaccination rate for a given time based on prodvided values for the vaccination start time and speed of rollout.

```
> rho.curve.ramp <- function(t, start = 100, speed = -0.08) {
+     ifelse(t <= start, rho <- 0, rho <- 0.96 * (1 - exp((t -
+         start) * speed)))
+     return(rho)
+ }
```

The direct method (Gillespie) is too slow for these parameters, so we use the adpativetau package for adpative tau leaping. Possible transitions and propensities (stochastic rates) are encoded as follows.

```
>   require(adaptivetau)
> transitions = cbind(c(1, 0, 0), c(-1, 0, 0), c(0, -1, 0), c(0,
+     0, -1), c(-1, 1, 0), c(0, -1, 1), c(0, 0, 1))
> transitions2 = cbind(c(1, 0, 0, 0), c(-1, 0, 0, 0), c(0, -1,
+     0, 0), c(0, 0, -1, 0), c(-1, 1, 0, 1), c(0, -1, 1, 0), c(0,
+     0, 1, 0))
> rates <- function(x, params, t) {
+     return(c(params$alpha + params$mu * (x["X"] + x["Y"] + x["Z"]),
+         params$mu * x["X"], params$mu * x["Y"], params$mu * x["Z"],
+         params$beta * x["X"] * x["Y"]/(x["X"] + x["Y"] + x["Z"]) +
+             params$xi * x["X"], params$gamma * x["Y"]))
+ }
> rates.vacc <- function(x, params, t) {
+     rho <- rho.curve.ramp(t, start = params$rho.start, speed = params$rho.speed)
+     return(c(params$alpha + params$mu * (x["X"] + x["Y"] + x["Z"]) *
+         (1 - rho), params$mu * x["X"], params$mu * x["Y"], params$mu *
+         x["Z"], params$beta * x["X"] * x["Y"]/(x["X"] + x["Y"] +
+         x["Z"]) + params$xi * x["X"], params$gamma * x["Y"],
+         params$mu * (x["X"] + x["Y"] + x["Z"]) * (rho)))
+ }
```

Model parameters are declared, grouped into a vector, and passed to the simulator.

```
> mu <- 1/60
> R0 <- 4
> gamma <- 365/12
> pop.size <- 1e+06
> beta <- R0 * (gamma + mu)
> ee0 <- (mu/beta) * pop.size * (R0 - 1)
> Y0 <- floor(ee0)
> Z0 <- floor(1 * (1 - 1/R0 - (mu/beta) * (R0 - 1)) * pop.size)
```

```
> X0 <- floor(pop.size - Y0 - Z0)
> params = list(gamma = gamma, beta = beta, mu = mu, xi = 0.001,
+       rho.start = 350, rho.speed = -0.05, alpha = 0)
> set.seed(10281979)
> r = ssa.adaptivetau(c(X = X0, Y = Y0, Z = Z0, W = 0), transitions2,
+       rates.vacc, params, tf = 450)
```

The following code produces Figure 1 of the main paper.

```
> par(mar = c(5, 4, 4, 4) + 0.1)
> matplot(r[, "time"], r[, c("Y")], type = "l", xlab = "Time",
+       ylab = "Prevalance (infected individuals)", col = "gray",
+       main = "Simulated smallpox dynamics during elimination")
> abline(h = ee0)
> p.star <- 1 - 1/R0
> t <- seq(0, 450)
> rho <- unlist(lapply(t, rho.curve.ramp, start = params$rho.start,
+       speed = params$rho.speed))
> crit <- t[which.min(rho < p.star)]
> R0v <- R0 * (1 - rho)
> ee <- ((mu/beta) * pop.size * (R0v - 1)) * (((mu/beta) * pop.size *
+       (R0v - 1)) > 0)
> lines(t, ee, col = ose1, lwd = 3)
> abline(v = crit, lty = 2)
> par(new = TRUE)
> plot(t, rho, xlab = "", ylab = "", xlim = par("usr")[1:2], xaxs = "i",
+       ylim = c(0, 1), type = "l", lwd = 3.5, axes = FALSE, col = "dodgerblue4")
> axis(4)
> mtext("Vaccination coverage", side = 4, line = 2.8)
```

Now we will look at some early warning signals. First we extract the number of new cases in each year using the custom function annualize to return periodically aggregated cases. The function pad.signal adds NA values as needed so we can use plotting functionality from the earlywarning package. The function plot.signal will produce the plot we want. Then we calculate moving window statistics across the "observed" epidemic time series. The timing of the critical point is shown with a vertical line.

```
> annualize <- function(r, period = 1) {
+       r <- data.frame(r)
+       r$year <- r$time%/%1
+       r$V <- c(0, diff(r$W))
+       data.annualized <- aggregate(r$V, by = list(r$year), FUN = sum)
+       names(data.annualized) <- c("year", "cases")
+       return(data.annualized)
+ }
> data.annual <- annualize(r)
> pad.signal <- function(x, ws) c(rep(NaN, (ws - 1)), x)
> plot.signal <- function(data, crit = NA, ws1 = 6, ws2 = 30, main = "",
+       axes = TRUE, xlab = "Time", ylab = "Coefficient of variation",
```

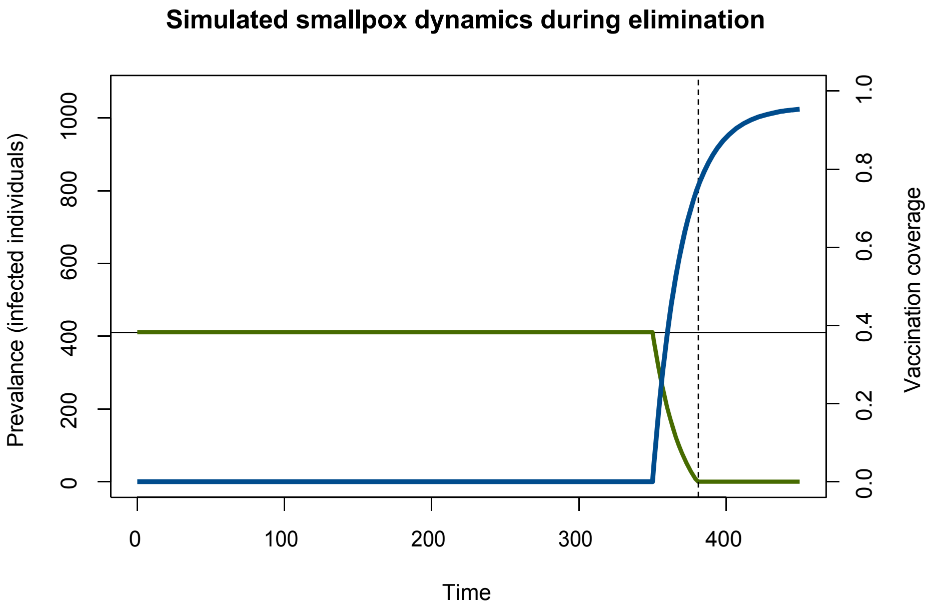

Figure 1: Simulation of a smallpox elimination campaign.

```
+       plot = TRUE) {
+       f <- exp(seq(1, ws1) * (log(0.01/1)/ws1))/sum(exp(seq(1,
+           ws1) * (log(0.01/1)/ws1)))
+       W1 <- filter(data[, 2], filter = f, sides = 1)
+       W2 <- pad.signal(sqrt(window_var(data[, 2], windowsize = ws2)),
+           ws2)/W1
+       if (plot == TRUE) {
+           plot(W2, log = "y", type = "l", xlab = xlab, ylab = ylab,
+               axes = axes, main = main)
+           abline(v = crit, lty = 2)
+       }
+       return(W2)
+ }
> par(mar = c(5, 4, 4, 4) + 0.1)
> plot(data.annual$year, data.annual$cases, type = "h", col = "grey",
+       ylab = "Cases", xlab = "Year")
> par(new = TRUE)
> ews <- plot.signal(data.annual, crit = crit, ws1 = 30, ws2 = 50,
+       main = "Statistical signal during the approach to elimination",
+       xlab = "", ylab = "", axes = FALSE)
> axis(4)
> mtext("Coefficient of variation", side = 4, line = 2.8)
> text(100, 0.5, labels = "i", cex = 1.8)
> text(350, 1, labels = "ii", cex = 1.8)
> text(430, 2.3, labels = "iii", cex = 1.8)
```

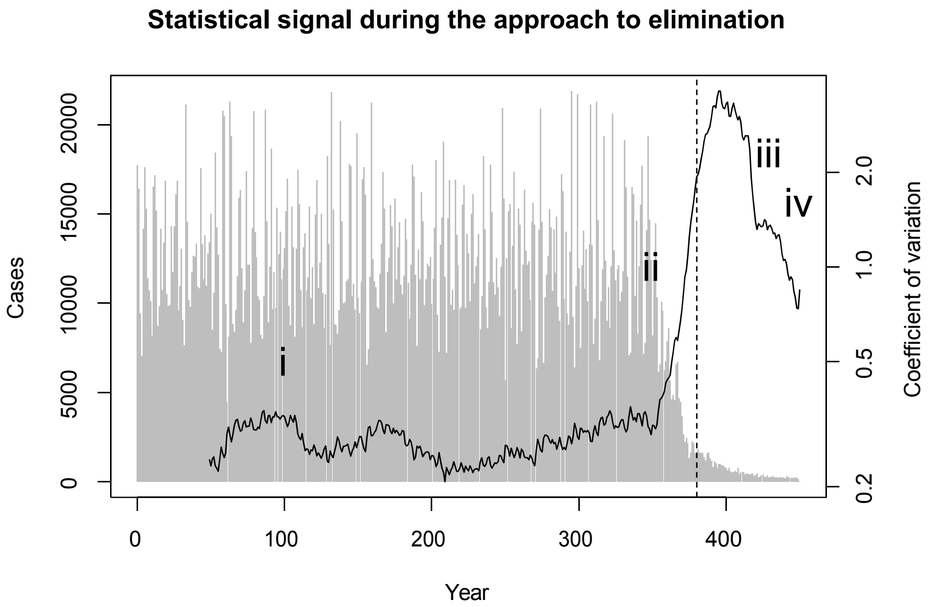

Figure 2: Early warning signals.

```
> text(450, 1.6, labels = "iv", cex = 1.8)
```

Here we look at how the signal is corrupted by binomially sampling the annualized incidence to represent underreporting. Evidently, the signal is quite robust to under-reporting.

```
> par(mfrow = c(5, 1))
> ews <- plot.signal(data.annual, crit = crit, ws1 = 30, ws2 = 50,
+      main = "Statistical signal during the approach to elimination")
> obs <- function(data, p = 0.95) data.frame(time = data$year,
+      cases = rbinom(seq(1, dim(data)[1]), size = data$cases, prob = p))
> ews95 <- plot.signal(w95 <- obs(data.annual, p = 0.95), crit = crit,
+      ws1 = 30, ws2 = 50, main = "95% Case Reporting")
> ews50 <- plot.signal(w50 <- obs(data.annual, p = 0.5), crit = crit,
+      ws1 = 30, ws2 = 50, main = "50% Case Reporting")
> ews10 <- plot.signal(w10 <- obs(data.annual, p = 0.1), crit = crit,
+      ws1 = 30, ws2 = 50, main = "10% Case Reporting")
> ews05 <- plot.signal(w01 <- obs(data.annual, p = 0.05), crit = crit,
+      ws1 = 30, ws2 = 50, main = "5% Case Reporting")
```

Estimating the tipping point

Finally, we look at some ways to estimate the threshold time. First, we seek a transformation to fit a hyperbolic model with linear regression. For instance, assuming the model $f_0 = y = -a/(a - c)$ and taking the inverse we have a linear equation $z = 1/y = (c - x)/a = c/a - x/a = -(1/a)x + c/a$.

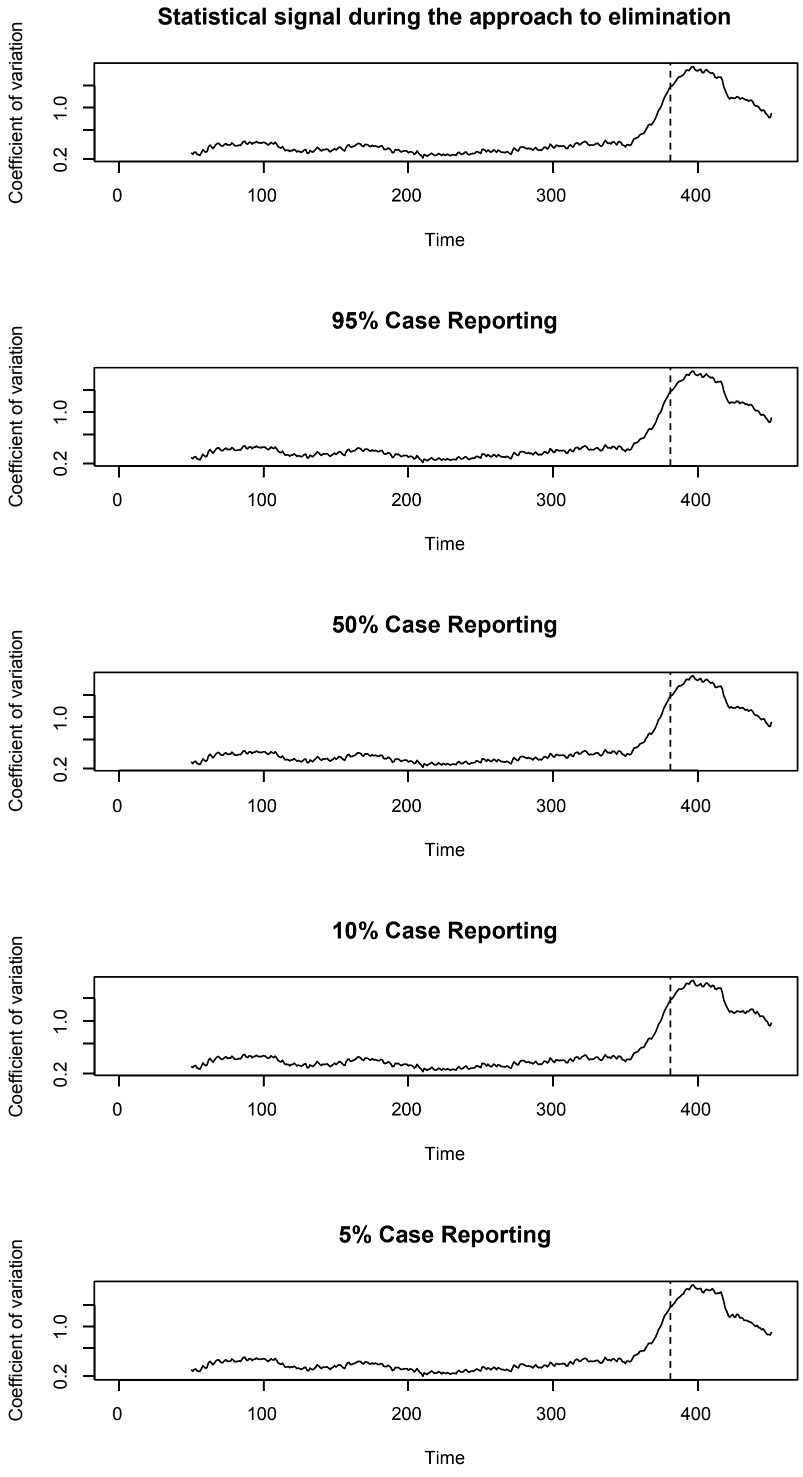

Figure 3: Early warning signals in time series corrupted by sampling.

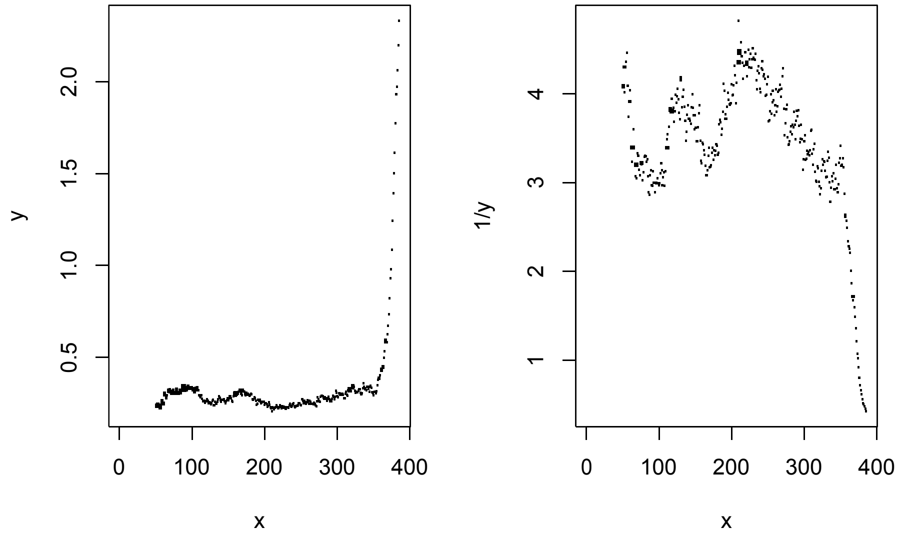

Figure 4: Transformation of a hyperbola model for the early warning signal.

Thus, taking the reciprocal of *y*, and regressing on *x*, the slope is the negative inverse of *a* and the intercept is *c/a* so that an estimator for the critical time is $c = -slope \times intercept$. He we try that with the early warning signal computed above.

```
> y <- ews[1:385]
> x <- 1:length(y)
> par(mfrow = c(1, 2))
> plot(x, y, type = "p", pch = ".")
> plot(x, 1/y, type = "p", pch = ".")
> z <- 1/y
> mod <- lm(z[200:385] ~ x[200:385])
> est <- -prod(coef(mod))
>  print(est)
```

```
[1] 0.132874
```

From Figure 4 we see that at least one problem with this approach is that the rectangular hyperbola $y = a/(x - c)$ assumes an asymptote at zero, which is a reasonable approximation on the original scale but not the inverse scale. Adding an asymptote we have $f_1 = y = a/(x - c) + b$, but this doesn't admit any tidy transformations.

Therefore, we switch to estimating the nonlinear function by least squares

```
> y <- ews[1:365]
> x <- 0:(length(y) - 1)
> plot(x, y)
> mod2 <- nls(y ~ -a/(x - c) + b, start = list(a = 10, b = 0.2,
```

```
+       c = 400))
>  print(mod2)

Nonlinear regression model
  model: y ~ -a/(x - c) + b
  data:  parent.frame()
       a        b        c
  1.3371   0.2677 368.8403
 residual sum-of-squares: 0.3274

Number of iterations to convergence: 7
Achieved convergence tolerance: 7.893e-06

> mod2.refit <- nls(y ~ -a/(x - c) + b, start = coef(mod2))
>   print(mod2.refit)

Nonlinear regression model
  model: y ~ -a/(x - c) + b
  data:  parent.frame()
       a        b        c
  1.3371   0.2677 368.8403
 residual sum-of-squares: 0.3274

Number of iterations to convergence: 0
Achieved convergence tolerance: 7.893e-06

> lines(x, -coef(mod2)[1]/(x - coef(mod2)[3]) + coef(mod2)[2])
```

Figure 5 shows this approach to work much better. In this example, the estimated critical time is 370 compared with a true value of 381. A weakness of this approach is that it can be expected to be numerically unstable so that if insufficient data are available or if start values for the fitting algorithm are pooly chosen it will run into difficulties.

The following function attempts to estimate the critical time directly from a signal such as that generated above.

```
> estimate.threshold <- function(ews, sig.time = NA, plot = FALSE,
+       a = 7.5) {
+       if (is.na(sig.time)) {
+           stop.50 <- 0.75 * max(ews, na.rm = TRUE)
+           sig.time <- min(which(ews > stop.50))
+       }
+       y <- ews[1:sig.time]
+       x <- 0:(length(y) - 1)
+       mod <- nls(y ~ -a/(x - c) + b, start = list(a = a, b = min(ews,
+           na.rm = TRUE), c = 1.05 * sig.time), control = c(max.iter = 500,
+           minFactor = 1/4096))
+       if (plot == TRUE) {
+           plot(x, y, pch = 16, cex = 0.5, col = "grey", xlab = "Time",
+               ylab = "Early warning signal", main = paste("Estimated threshold:",
```

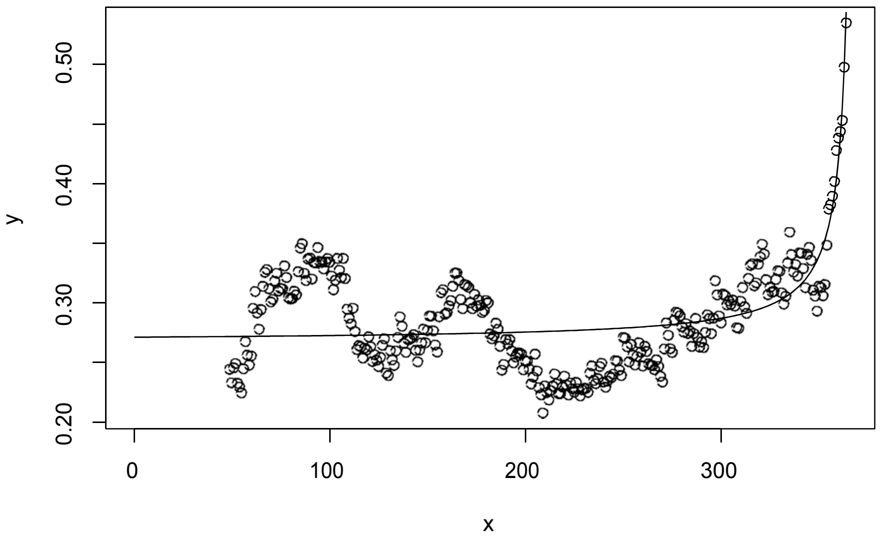

Figure 5: Nonlinear least squares fit to a hyperbola model for the early warning signal.

```
+                       round(unname(coef(mod))[3])))
+           lines(x, -coef(mod)[1]/(x - coef(mod)[3]) + coef(mod)[2],
+               lwd = 2, col = "dodgerblue4")
+       }
+         return(estimated.threshold <- unname(coef(mod))[3])
+ }
```

It's use is demonstrated as follows:

```
> threshold <- estimate.threshold(ews, plot = TRUE)
```

The result is shown in Figure 6.

What might we compare these estimates to? One proposal is simple extrapolation from the observed trend. Of course, this doesn't predict the tipping point, just the extinction time. But, if bifurcatioin delay is small then these should be close. Our extrapolating estimator will perform the following steps:

1. Select that portion of the incidence data from the last the number of cases was greater than 90% of its maximum to some later time at which the extrapolation is to be made.
2. Log transform the data and fit a linear equation: $log(w + 1) = mx + b$, where w is annual incidence (one is added to observations particularly to faclitate studies of under-reporting, which will often yield values of zero).
3. Solve the resulting equation for $log(w + 1) = 0$ yielding estimator $\hat{t} = -b/m$ for the critical time.

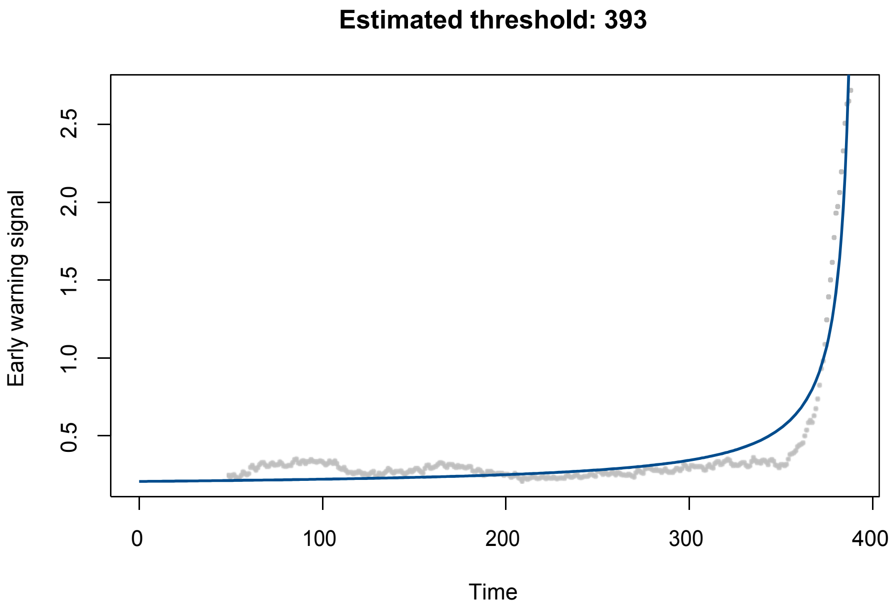

Figure 6: Demonstration of the use of the threshold estimator.

```
> extrapolator <- function(data, sig.time = NA, plot = FALSE) {
+       if (is.na(sig.time))
+           sig.time <- length(data)
+       ma <- max(data, na.rm = TRUE)
+       ma90 <- 0.9 * ma
+       w <- max(which(data > ma90))
+       y <- data[w:sig.time]
+       x <- (w - 1):(sig.time - 1)
+       z <- log(y + 1)
+       mod <- lm(z ~ x)
+       if (plot) {
+           plot(x, z, xlab = "Time", ylab = "Cases", main = paste("Estimated threshold:",
+               round(unname(-coef(mod)[1]/coef(mod)[2]))))
+           abline(mod, lwd = 2, col = "dodgerblue4")
+       }
+       return(extrapolated.threshold <- unname(-coef(mod)[1]/coef(mod)[2]))
+ }
```

This function is demonstrated as follows and illustrated in Figure 7

```
> extrapolated.threshold <- extrapolator(data.annual$cases, plot = TRUE)
```

Now, we'd like to see how the bias and variance of the the early warning statistic and extrapolator estimate change with under-reporting in $n = 1000$ simulations.

```
> n.sims <- 1000
```

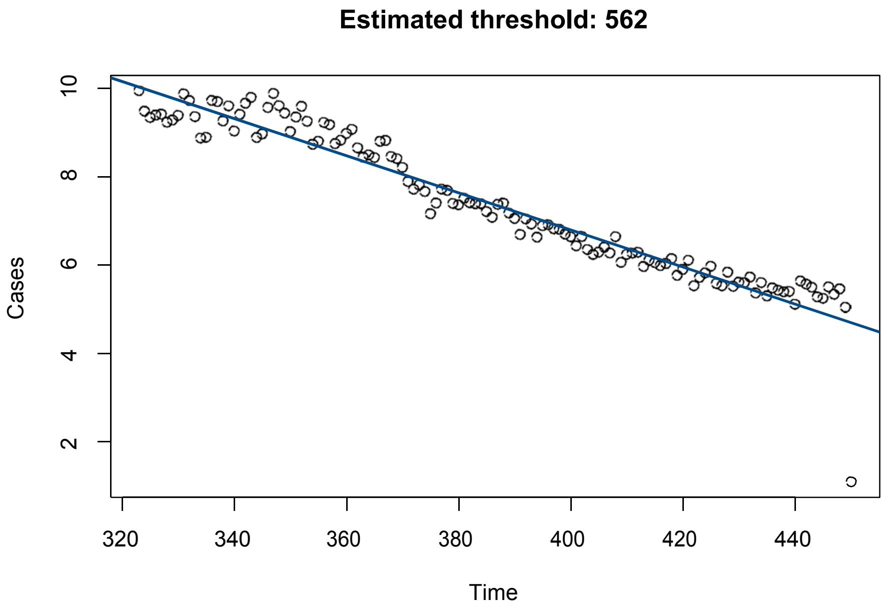

Figure 7: Demonstration of the use of an extrapolation approach to estimating the critical threshold.

```
> sims <- lapply(1:n.sims, function(x) ssa.adaptivetau(c(X = X0,
+     Y = Y0, Z = Z0, W = 0), transitions2, rates.vacc, params,
+     tf = 450))
> save(sims, n.sims, file = "sims.RData")

> load("sims.RData")
> sims.annualized <- rapply(sims, f = function(x) annualize(x),
+     how = "list")
> set.seed(10281979)
> sims95 <- lapply(sims.annualized, function(x) obs(x, p = 0.95))
> sims50 <- lapply(sims.annualized, function(x) obs(x, p = 0.5))
> sims10 <- lapply(sims.annualized, function(x) obs(x, p = 0.1))
> sims05 <- lapply(sims.annualized, function(x) obs(x, p = 0.05))
> thresh <- unlist(lapply(sims.annualized, FUN = function(x) extrapolator(x$cases)))
> thresh95 <- unlist(lapply(sims95, FUN = function(x) extrapolator(x$cases)))
> thresh50 <- unlist(lapply(sims50, FUN = function(x) extrapolator(x$cases)))
> thresh10 <- unlist(lapply(sims10, FUN = function(x) extrapolator(x$cases)))
> thresh05 <- unlist(lapply(sims05, FUN = function(x) extrapolator(x$cases)))
> sims.ews <- lapply(sims.annualized, FUN = function(x) plot.signal(x,
+     plot = FALSE))
> sims95.ews <- lapply(sims95, FUN = function(x) plot.signal(x,
+     plot = FALSE))
> sims50.ews <- lapply(sims50, FUN = function(x) plot.signal(x,
+     plot = FALSE))
> sims10.ews <- lapply(sims10, FUN = function(x) plot.signal(x,
```

```
+       plot = FALSE))
> sims05.ews <- lapply(sims05, FUN = function(x) plot.signal(x,
+       plot = FALSE))
> thresh.ews <- unlist(lapply(sims.ews, FUN = function(x) estimate.threshold(x)))
> thresh95.ews <- unlist(lapply(sims95.ews, FUN = function(x) estimate.threshold(x)))
> thresh50.ews <- unlist(lapply(sims50.ews, FUN = function(x) estimate.threshold(x)))
> thresh10.ews <- unlist(lapply(sims10.ews, FUN = function(x) estimate.threshold(x)))
> thresh05.ews <- unlist(lapply(sims05.ews, FUN = function(x) estimate.threshold(x)))
> threshold <- data.frame(threshold = c(thresh.ews, thresh95.ews,
+       thresh50.ews, thresh10.ews, thresh05.ews, thresh, thresh95,
+       thresh50, thresh10, thresh05), reporting = rep(c(rep(100,
+       n.sims), rep(95, n.sims), rep(50, n.sims), rep(10, n.sims),
+       rep(5, n.sims)), 2), method = c(rep("ews", n.sims * 5), rep("extrapolation",
+       n.sims * 5)))
> boxplot(threshold ~ method + reporting, data = threshold, col = rep(c("dodgerblue4",
+       ose1), 5), axes = FALSE, log = "", ylim = c(350, 1500))
> axis(2)
> axis(1, at = seq(0, 8, by = 2) + 1.5, labels = c("5%", "10%",
+       "50%", "95%", "100%"))
> box()
> legend("topleft", bty = "n", col = c("dodgerblue4", ose1), legend = c("Critical slowing down
+       "Extrapolation"), lty = 1, lwd = 6)
> save(sims.annualized, sims95, sims50, sims10, sims05, thresh,
+       thresh95, thresh50, thresh10, thresh05, sims.ews, sims95.ews,
+       sims50.ews, sims10.ews, sims05.ews, thresh.ews, thresh95.ews,
+       thresh50.ews, thresh10.ews, thresh05.ews, threshold, file = "bias.RData")
```

Of course, since the extrapolator has no information about bifurcation delay it will inevitably be very biased. What's more, there's no "natural" way to correct for this bias. Therefore, we will draw a plot showing just the effect of under-reporting on the critical slowing down based estimator.

```
> load("bias.RData")
> par(mfrow = c(1, 2))
> par(mar = c(5, 4, 2, 0) + 0.1)
> plot(sims.ews[[1]], lwd = 2, col = "dodgerblue4", ylab = "Coefficient of variation",
+       main = "(a) Under-reporting")
> lines(sims95.ews[[1]], lwd = 2, col = ose1)
> lines(sims50.ews[[1]], lwd = 2, col = ose2)
> lines(sims10.ews[[1]], lwd = 2, col = ose3)
> lines(sims05.ews[[1]], lwd = 2, col = ose4)
> legend("topleft", legend = c("100% Reporting", "95% Reporting",
+       "50% Reporting", "10% Reporting", "5% Reporting"), col = c("dodgerblue4",
+       ose1, ose2, ose3, ose4), lwd = 2, bty = "n")
> boxplot(threshold ~ method + reporting, data = threshold[threshold$method ==
+       "ews", ], col = rep(c("dodgerblue4", ose1), 5), axes = FALSE,
+       ylim = c(360, 410), xlab = "Fraction of cases reported",
+       ylab = "Estimated threshold", main = "(b) Bias")
> axis(2)
```

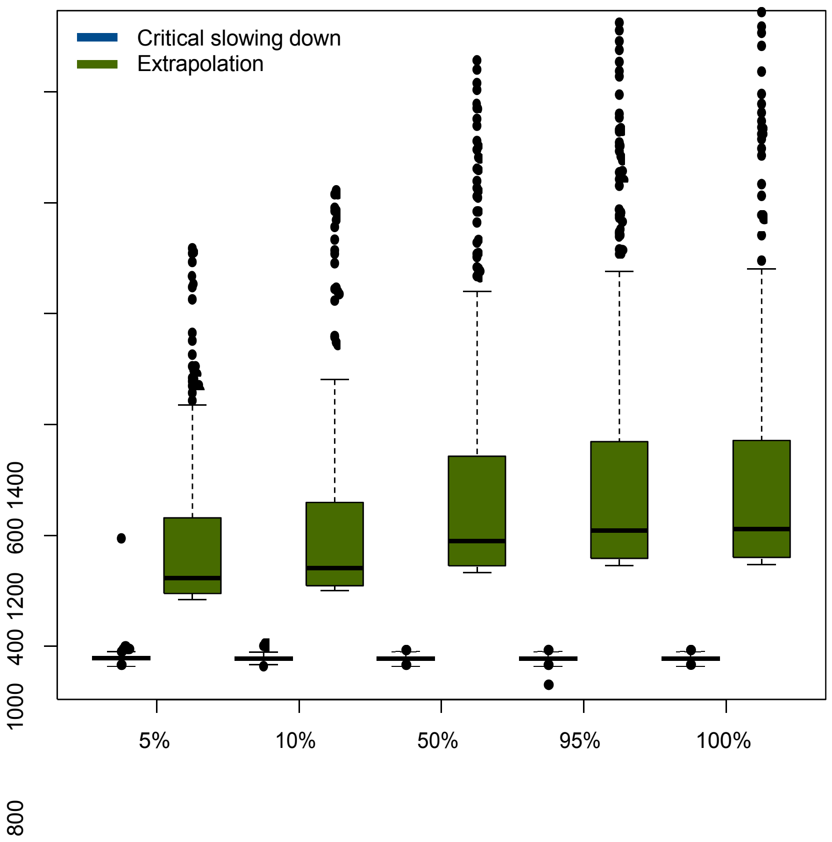

Figure 8: Comparison of the extrapolation and critical slowing down based approaches to estimating the critical threshold.

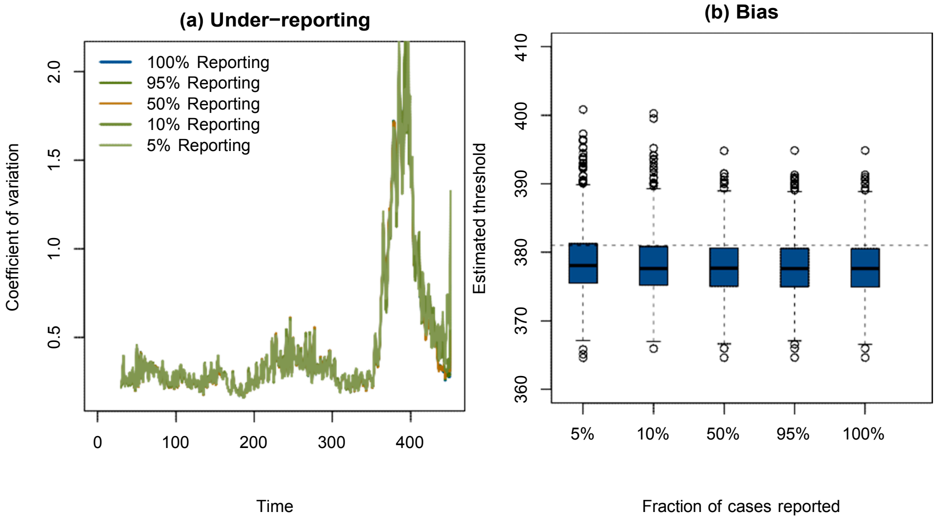

Figure 9: Bias in an approach to estimating the critical threshold from measurement of critical slowing down. Note that lines in the left panel are largely, but not exactly, overlapping.

```
> axis(1, at = seq(0, 8, by = 2) + 1, labels = c("5%", "10%", "50%",
+      "95%", "100%"))
> box()
> abline(h = crit, lty = 2)
```

Figure 9 shows that there is a small amount of negative bias in this critical slowing down estimator of the critical point (the threshold is predicted to be earlier than than it actually is). One thing I've found is that this is somewhat sensitive to the time at which the prediction is made. Possibly this is also due to the size of the moving window used for calculating the variance. In this experiment this is set to 30 observations. A smaller moving window would have less inertia (would adjust faster), but preliminary numerical experiments found this to trade off with the estimability of a in the hyperbola model: with fewer than about 30 observations in the window it was not possible to find starting positions for a that would actually converge. Some additional experiments showed that models could be fit with reporting as low as 1% or 2% but automated fitting was impossible and the variance in the estimate became very large (though not as large as for the extrapolation method).
